# Differentiation of Body Fluid Stains Using a Portable, Low-Cost Ion Mobility Spectrometry Device—A Pilot Study

**DOI:** 10.3390/molecules28186533

**Published:** 2023-09-09

**Authors:** Cameron Heaton, Simon Clement, Paul F. Kelly, Roberto S. P. King, James C. Reynolds

**Affiliations:** 1 Foster + Freeman, Evesham WR11 1TD, UK; simon.clement@fosterfreeman.com (S.C.); roberto.king@fosterfreeman.com (R.S.P.K.); 2 Department of Chemistry, Loughborough University, Loughborough LE11 3TU, UK; p.f.kelly@lboro.ac.uk (P.F.K.); j.c.reynolds@lboro.ac.uk (J.C.R.)

**Keywords:** IMS, ion mobility spectrometry, blood, urine, biofluids, forensics, detection

## Abstract

The identification and recovery of suspected human biofluid evidence can present a bottleneck in the crime scene investigation workflow. Crime Scene Investigators typically deploy one of a number of presumptive enhancement reagents, depending on what they perceive an analyte to be; the selection of this reagent is largely based on the context of suspected evidence and their professional experience. Positively identified samples are then recovered to a forensic laboratory where confirmatory testing is carried out by large lab-based instruments, such as through mass-spectrometry-based techniques. This work proposes a proof-of-concept study into the use of a small, robust and portable ion mobility spectrometry device that can analyse samples in situ, detecting, identifying and discriminating commonly encountered body fluids from interferences. This analysis exploits the detection and identification of characteristic volatile organic compounds generated by gentle heating, at ambient temperature and pressure, and categorises samples using machine learning, providing investigators with instant identification. The device is shown to be capable of producing characteristic mobility spectra using a dual micro disc pump configuration which separates blood and urine from three visually similar interferences using an unsupervised PCA model with no misclassified samples. The device has the potential to reduce the need for potentially contaminating and destructive presumptive tests, and address the bottleneck created by the time-consuming and laborious detection, recovery and analysis workflow currently employed.

## 1. Introduction

Biological samples can be invaluable in criminal investigations; the presence of blood can indicate a violent altercation, semen can indicate sexual activity, and the presence of saliva, sweat and urine can indicate the presence of individual/s in a certain location. Some of these fluids may also yield DNA, which can be matched against a database, or a suspect sample, for identification/exoneration purposes. However, depending on the circumstances of the crime, it might not be immediately obvious which body fluid a Crime Scene Investigator (CSI) has encountered. Biofluid traces may look similar to one another, depending on varying factors such as age, quantity and deposition substrate, or because they have actually encountered a non-biofluid contaminant trace that may or may not be relevant to the circumstances surrounding the crime.

CSIs typically rely on colorimetric presumptive tests to indicate the presence of a certain biofluid at the scene. Different reagents exist for each biofluid, and investigators must make an assumption of the most likely type of biofluid evidence before applying the appropriate test reagent. The application of these reagents can contaminate the samples, and will also lower the concentration of analytes by dilution. They can also be prone to false-positive and false-negative results, depending on the specificity of their chemical mechanism of action, by both opposing biofluids [1] and unrelated substances [2]. Some need to be viewed under specialist conditions, for example, the use of Luminol for the presumptive detection of blood is best observed in the dark with highly sensitive camera equipment, as the chemiluminescence is weak and short lived. Reagents can also be hazardous to operators and destructive to certain substrates; for example, a water-based formulation of Acid Black 1 was proposed by the Home Office rather than a methanol-based formulation. This was due to concerns about methanol’s toxicity and flammability, and its ability to “soften or destroy some surfaces including paints, varnishes and some plastics”, despite methanol being reported as the superior fixing agent [3]. Reagents can also have limited shelf-lives. The Home Office recommends that acid dye reagents are stored for no longer than 12 months at room temperature [4].

Stains that have yielded a positive colour change from a presumptive test must then be recovered (usually via a destructive swabbing technique) and have the initial presumptive identification corroborated by a confirmatory laboratory test [5]. These are commonly mass-spectrometry-based techniques, requiring sample preparation steps such as reconstitution and chromatographic separation. This process of being tested twice is time consuming for the investigation, prolonging the time during which evidence of value can be acted upon. Furthermore, this introduces a delay between the initial recovery of evidence and confirmatory analysis; over this time, sample degradation may occur. The recent under-resourcing of the forensic sector can lead to backlogs between sample submission and the reporting of results [6], which can allow serial offenders time to reoffend, or can delay the exoneration of suspects held in custody. Any delay in processing evidence is a particular concern for samples waiting to undergo DNA analysis, where severe degradation can mean that insufficient material is available for successful profiling. However, due to the time, costs and complexity associated with DNA profiling, it is important to definitively confirm that suspected stains are in fact biofluids before submission for DNA analysis.

While blood and semen both contain a very high concentration of cells and are therefore good sources of DNA, saliva, sweat and urine also have the potential to yield DNA profiles [7,8,9,10,11,12,13]. The ability to detect these evidential bodily fluids in situ, distinguishing them from contaminants (both visually similar and those that yield false positives in response to currently used presumptive tests) whilst providing confirmatory identification at the crime scene, would critically speed up the current detection and identification workflow. This would enable samples to be submitted for DNA analysis sooner, minimising the chance of sample degradation, and reducing the time between sample recovery and result generation. To advance the development of an in situ analyser, this study has employed thermal desorption as a method of sample introduction, coupled to an ion mobility spectrometry (IMS) instrument.

Thermal desorption is an established technique used to generate vapour phase analytes, which can be coupled to a range of analytical instruments. Mass spectrometers are commonly coupled with volatile organic compound (VOC) samplers to analyse a variety of forensic sample types such as biofluids, drugs [14,15], explosives [16,17,18] and fire debris [19]. Mass spectrometric approaches can also be used in combination with ion mobility spectrometry (IMS) and have been used to measure explosives [20] and drugs [15,21], where the IMS offers an additional dimension of separation, able to resolve species with identical molecular masses, but differing collisional cross sections (CCS). Configurations utilising mass spectrometry are usually large and not portable, and do not lend themselves to in situ analysis. Although atmospheric pressure mass spectrometry instruments are available, many high-resolution instruments require the sample to be under vacuum, presenting a challenge to the potential portability of these units. IMS, in contrast, can be operated at ambient pressure. This, combined with its high sensitivity, fast response time and relatively simple design, gives IMS devices greater potential to be used in the field.

IMS is a technique currently employed for other forensic and security applications and has historically been used for the detection of volatile compounds spanning several forensic applications, including the identification of explosives [22,23], drugs [22,23] and chemical weapons [23]. Static scanning devices are commonly employed at airports and borders [24] to aid in the detection of threats (explosives) and contraband (drugs). IMS is also the most common technology utilised in handheld chemical warfare agent (CWA) detection devices [25]. However, to the best of the authors’ knowledge, research has not previously reported on the use of IMS for the forensic detection of body fluids, particularly under ambient conditions.

This study explores the feasibility of using contactless thermal desorption combined with IMS as a rapid portable method to detect and identify a subset of body fluids and contaminants and overcome some of the disadvantages associated with colorimetric reagent tests.

## 2. Results and Discussion

### 2.1. Venturi Pump Configuration

**Figure 1:**
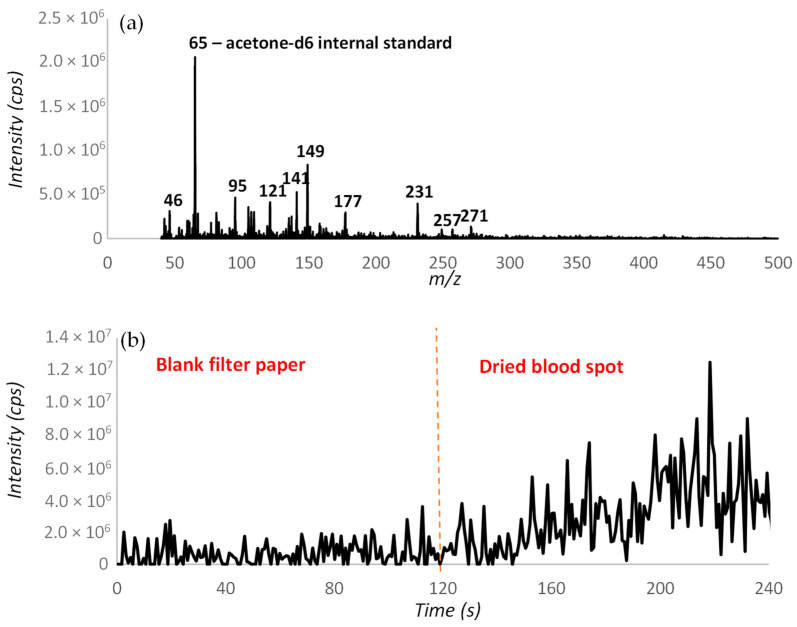
(**a**) Background-subtracted mass spectrum obtained from a defibrinated horse blood sample using DOS-APCI-MS (**b**) extracted ion trace for *m*/*z* 231.

The desorption off surface (DOS) probe assembly developed by Rankin-Turner et al. [26] uses a heated gas flow to thermally desorb volatile organic compounds from solidified sample deposits before extraction into the mass spectrometer using a Venturi jet pump. An analysis of 1-day-old, dried, defibrinated horse blood samples deposited on filter paper was conducted by measuring background interferences from blank filter paper for 2 min, before shifting the probe to the dried blood spot for a further 2 min to enable background subtraction. This analysis yielded the background-subtracted mass spectrum shown in Figure 1a, which shows that a range of different mass spectral peaks were detected from the dried, defibrinated blood spots. Components which arise from the dried blood spot can then be confirmed by checking their signal response in the background and dried blood spot. Figure 1b shows the response for *m*/*z* 231 which is seen to increase when the probe is moved onto the dried blood spot at the 2 min time point.

**Figure 2:**
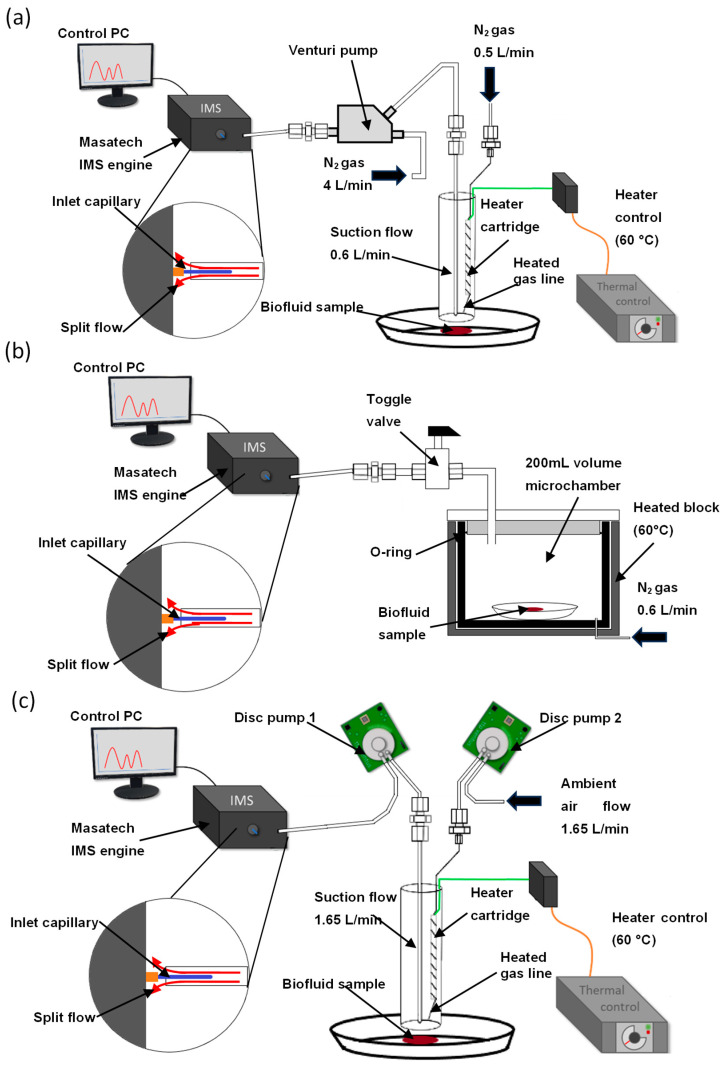
Instrumental configurations (not to scale). (**a**) Desorption off surface (DOS) probe AIMS interface with Venturi pump, split flow inlet shown in inset, (**b**) microchamber thermal extractor interface and (**c**) dual-disc pump DOS probe AIMS configuration.

Having demonstrated that the probe assembly could successfully extract thermally desorbed VOCs from a sample surface for MS analysis, the DOS probe configuration was then coupled directly to the Masatech AIMS system using the same Venturi jet pump system as that used in reference [26] (Figure 2a). However, when coupled to IMS, this instrumental configuration was not capable of detecting volatile organic compounds from defibrinated horse blood samples.

The main issue which limited the performance of the DOS-IMS system was found to be the dilution of analytes in the desorption gas flow and subsequent loss through flow splitting to reach a flow compatible with the IMS. The mass spectrometer described in reference [26] uses the nebuliser gas flow for the APCI source (4 L/min) as the gas flow through the Venturi pump. This flow is required to generate the 0.6 L/min suction flow which extracts the thermally desorbed analytes from the surface under investigation. Extracted analytes are diluted in this flow, reducing their gas-phase concentration. In the APCI-MS system, all of the combined 4.6 L/min gas flow enters the ion source. However, this does not occur with the IMS system, which draws in gas at a continuous rate of ~0.2 L/min through a narrow PEEK capillary tube. To interface the combined 4.6 L/min flow from the Venturi pump a split inlet was constructed to avoid over-pressuring and damaging the IMS. This inlet enabled the IMS to sample from the gas flow exiting the Venturi pump, while the remainder of the flow was exhausted. These experiments showed that with the dilution factor from the use of the Venturi pump, and the loss of approximately 95% of the desorbed analytes through the split, the Venturi pump configuration lacks the requisite sensitivity for biofluid analysis from surfaces when combined with the IMS system and thus an alternative approach is required.

### 2.2. Microchamber Thermal Extractor Configuration

To address these issues and produce a detectable signal that would persist long enough to enable the optimisation of instrument parameters, a configuration using a Markes µCTE-250 microchamber was assembled (Figure 2b). The microchamber allowed a low flow rate of gas (600 mL/min) to be passed through a heated 200 cm^3^ volume silcosteel chamber and directed via a manual switching valve to the IMS inlet capillary. This system removed dilution due to the Venturi pump and thus produced a more concentrated analyte vapour. In addition, for the purpose of optimisation, it also eliminated variation due to the position of the DOS probe with respect to the surface and since the flow through the split inlet was lower, it enabled a larger proportion of the analyte to enter the ion source of the IMS.

**Figure 3:**
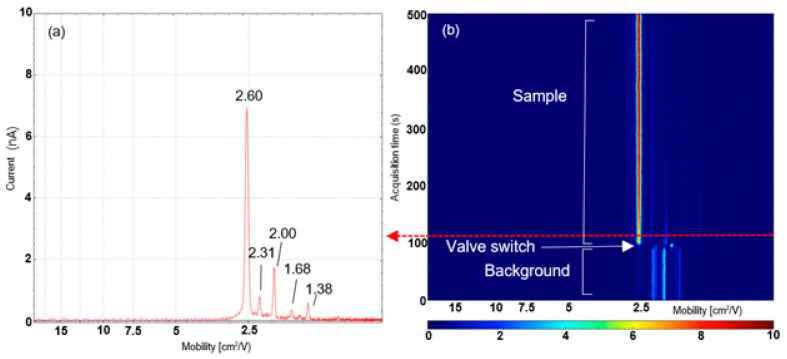
Ion mobility data from a 10 µL dried defibrinated horse blood spot obtained using the microchamber thermal extractor configuration. (**a**) IMS profile taken at 110 s when sampling from the dried blood spot. (**b**) A 3D heat map showing mobility versus time, indicating switching between background and dried blood spot profiles.

Using the microchamber thermal extractor configuration allowed a dried analyte sample to be introduced into the chamber and heated gently with the valve closed to allow VOCs to collect in the headspace above the sample; when the valve is opened, the concentrated headspace is then extracted as a slug into the mass spectrometer ion source by the gas flow. Using this configuration, distinctive IMS profiles of biofluids were obtained. An example 2D-IMS heat map obtained from a defibrinated horse blood sample using the microchamber configuration is shown in Figure 3, which shows a 5 min analysis of a defibrinated horse blood sample. For the first 90 s of this analysis, the valve is in the closed position which enables a background spectrum to be obtained from the system. The ion mobility spectrum of the background shows a number of different peaks; the peak at 2.19 cm^2^/Vs is the reactant ion peak (RIP) which is composed of protonated water clusters of the form [(H_2_O)_n_ H^+^]^+^. In addition a number of other peaks at a lower intensity are noted in the background spectrum at 1.74, 1.82, 2.05, 2.25 and 2.62 cm^2^/Vs, respectively. The identity of these species cannot be confirmed with the IMS alone; however, they are most likely to originate from solvent vapours and other trace atmospheric constituents in the laboratory environment.

When the valve is switched at 90 s, the headspace from the microchamber is directed into the IMS. Figure 3a shows the IMS trace obtained at ~110 s after, when the profile from the defibrinated blood is observed. The RIP in this trace is fully depleted and five new peaks appear in the trace at 1.38, 1.68, 2.00, 2.31 and 2.60 cm^2^/Vs, respectively, which are present for two or more minutes of the sample analysis, indicating they are continuously emitted from the solid sample during analysis. All of these peaks, except the intense peak at 2.62 cm^2^/Vs, decrease in intensity throughout the analysis, indicating they are being gradually exhausted from the sample. This is not observed with the peak at 2.62 cm^2^/Vs, which indicates it is present at high levels in the sample or may be formed by other species thermally degrading. This peak is most likely ammonia, which is also observed at low levels in the background spectrum. Ammonia is known to be present in horse blood at levels of around 40 µM, so its presence in the IMS spectra is expected [27]; it may also be released from other species such as amino acids or proteins due to thermal degradation during heating.

An additional intense peak is also detected at 1.80 cm^2^/Vs. This species is very quickly washed out from the system, indicating this is either a highly volatile analyte which has partitioned almost entirely into the headspace during equilibration or is a background species introduced by the operation of the valve. In addition to blood, two other biofluids (saliva and urine) were analysed using this configuration (Figures S1 and S2). Both biofluids produced characteristic ion mobility spectra, which were distinct enough from each other to enable discrimination between the three biofluids tested. Using this configuration, it was possible to conduct experiments to determine how sensitive the system is. Characteristic IMS spectra for each of the three biofluids were obtained from as little as 1 µL of deposited material. This demonstrated that the microchamber IMS system is capable of detecting biofluids at forensically relevant levels. A typical single drop of blood or other aqueous solution will contain approximately 20–25 µL of fluid, demonstrating that even small traces can be characterised with the system.

The data obtained from the microchamber thermal extractor showed that by removing the dilution factor associated with the Venturi pump, the IMS was capable of reliably detecting and discriminating biofluids from microlitre volume droplets with this configuration. However, this system has a number of drawbacks which would preclude its use in the field. The microchamber itself is large and bulky and requires a flow of nitrogen/air to extract volatiles from the chamber. While it could be miniaturised in theory, its biggest limitation is that once a chamber has been used for an analysis, it takes a considerable amount of time (in the region of 30 min) for VOCs to be extracted from the chamber before it can be used again. This raises the possibility of cross contamination and memory effects between samples, which could result in false positive test results as well as significantly reduce sample throughput, thus limiting its application as a rapid screening tool.

### 2.3. Dual-Disc Pump Configuration

To address the issues with the previous two configurations, a dual-disc pump configuration for the DOS probe was developed (Figure 2c). The use of the disc pumps removes the need for a gas flow to the probe as both pumps will supply compressed air to the probe, with the IMS running on compressed air supplied by a built-in pump; this configuration gives a portable system which is not reliant on an external gas supply. The disc pumps are balanced to provide a 1.65 L/min flow each; this reduces the dilution of the sample observed with the Venturi pump configuration while maintaining the ability to take samples rapidly in situ, with short clearance times between samples.

**Figure 4:**
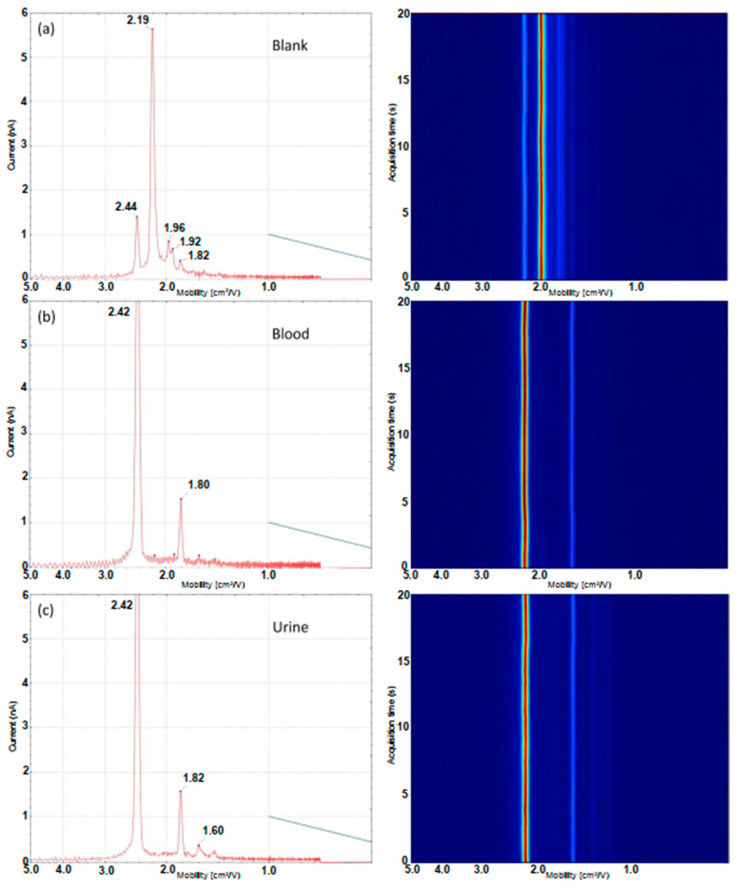
Ion mobility traces (mobility vs. ion current) and 3D drift time heat map (mobility vs. time) for a blank (**a**) and 2 × 10 µL dried biofluid samples; (**b**) blood and (**c**) urine obtained using the dual-disc pump–probe configuration.

**Figure 5:**
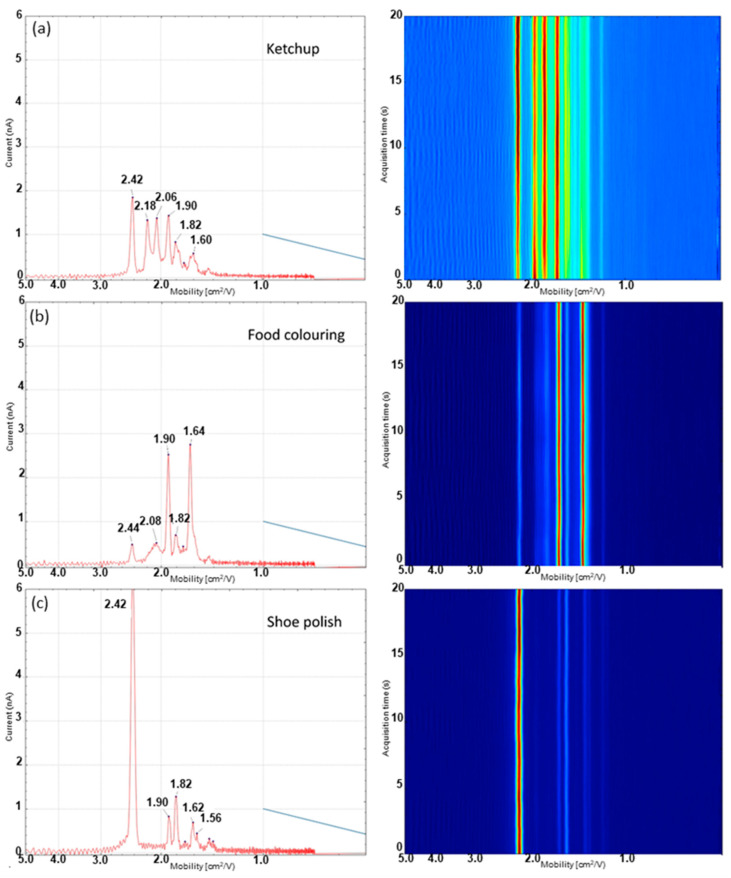
Ion mobility traces (mobility vs. ion current) and 3D drift time heat map (mobility vs. time) for 3 visually similar interferants. (**a**) Ketchup, (**b**) food colouring and (**c**) shoe polish obtained using the dual-disc pump–probe configuration.

The dual-disc pump configuration was applied to study blood, urine and three common contaminants which show a similar visual response under polarised light. Using the disc pump configuration, characteristic IMS profiles for each substance were obtained which were sufficiently unique that when combined with statistical processing, the system was capable of reliably differentiating each of the fluids and interferences from each other. The averaged ion mobility traces and three dimensional heatmaps showing ion mobility drift time values over a 20 s acquisition period are shown in Figure 4 and Figure 5. A video of the real-time sample acquisition of each of the samples is available in the Supplementary Materials (Video S1).

As with the microchamber configuration, the predominant peak present in the blank (Figure 4a) at a mobility value of 2.19 cm^2^/Vs is the reactant ion peak (RIP). The smaller peak present at a mobility value of 2.44 cm^2^/Vs is attributed to background ammonia. It is worth noting this appears at a slightly lower mobility value than what is observed in the microchamber experiments. This effect occurs most likely due to the use of ambient air rather than dried nitrogen as the carrier gas; ambient air will result in neutral water molecules clustering with the analyte ions and result in a shift to lower mobility due to the increased collision cross-section of the ion–molecule cluster. In addition to these two species, there are three other low-intensity responses observed at 1.82, 1.92 and 1.96 cm^2^/Vs, respectively. When analysing both blood and urine samples, an intense signal is observed for ammonia which is expected to be present at high levels in both blood and urine samples. The profile obtained using the dual-disc pump–probe configuration from the dried defibrinated blood spot (Figure 4b) did not show as much detail as the microchamber configuration analysis of defibrinated horse blood with only two characteristic ion responses observed. These peaks corresponded to the two most intense responses detected using the microchamber configuration, the ammonia peak at 2.42 cm^2^/Vs and a second less intense peak at 1.80 cm^2^/Vs. The other responses likely fall below the limit-of-detection and are not reliably detected. The loss in sensitivity compared to the microchamber interface occurs most likely due to the loss of thermally desorbed VOCs to the environment in the probe interface. The urine profile also shows ion responses at the same drift times as blood. However, another peak at 1.60 cm^2^/Vs was detected in urine spots which was not observed in blood spots, which enables discrimination. A full list of peaks detected in both biofluids, blanks and interferences is shown in Table 1 for comparison.

The results from the analysis of three common interferants, which show visually similar responses to blood under polarised light, are shown in Figure 5. Both ketchup (Figure 5a) and food colouring (Figure 5e) show dramatically different IMS spectra from both of the dried biofluid spots. The spectra of these two interferants show a range of different peaks which are not observed in the spectra of either of the two biofluids, making the discrimination of the biofluids from the interferences possible. Shoe polish (Figure 5c) shows more similarity to the biofluid samples with an intense response detected for ammonia at 2.42 cm^2^/Vs. However, it also shows two peaks which were not present in either of the biofluids tested at 1.90 and 1.56 cm^2^/Vs, respectively.

**Figure 6:**
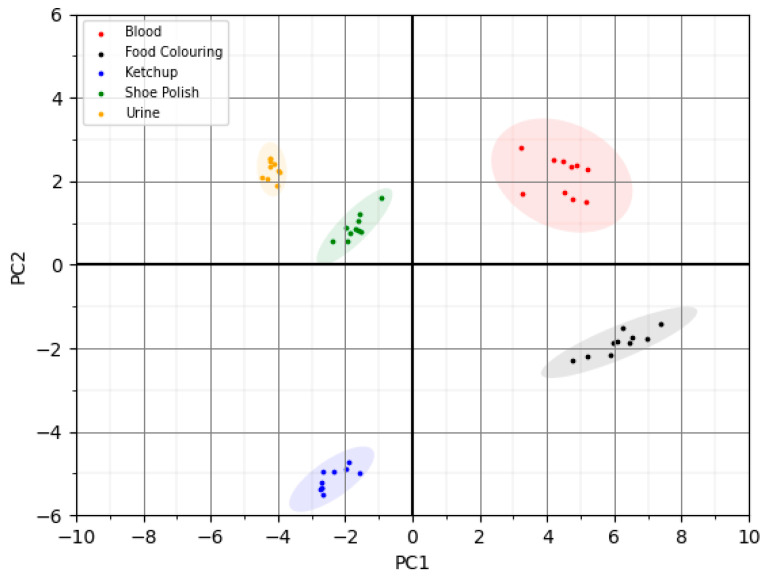
PCA plot of blood, urine and visually similar contaminants.

The data were then input into the built-in Masatech Chemometrics package and an unsupervised principal component analysis method was used to develop a classification model. Ten replicate measurements of each biofluid and interferants were used to create the model, which is shown in Figure 6. The confidence ellipses around the points show a difference of three standard deviations from the mean, indicating complete separation of the different biofluids and interferences using an unsupervised model with no misclassified samples. The ellipses show good grouping of the replicate measurements indicating that despite the open nature of the probe interface for surface sampling, it is possible to obtain reliable data in this lab-based scenario with little evidence of inter-sample variation.

## 3. Materials and Methods

### 3.1. Materials

Defibrinated horse blood was obtained from TCS Biosciences Ltd. (Buckingham, UK). Saliva and urine were obtained directly from the investigators. Black shoe polish (KIWI^®^, S. C. Johnson, Denby, UK), black food colouring (Sainsbury’s, London, UK) and tomato ketchup (Lichfields, London, UK) were purchased from a local supermarket. These 3 substances were used as they appear visually similar to dried blood when dried and when investigated using a polarised light source. Aluminium dishes were purchased from Sigma Aldrich (Gillingham, UK).

### 3.2. Sample Preparation

Sample preparation for mass spectrometry analysis was conducted by spotting ~25 µL of defibrinated horse blood on Whatman 0.45 µm filter paper and allowing it to air dry. Samples for IMS analysis, including biofluids and visually similar contaminants, were deposited in separate aluminium dishes and allowed to fully dry at room temperature. Ten replicates of each substance were produced. For comparative studies, 10 µL droplets of each biofluid and interference were used.

### 3.3. IMS and Interface Conditions

#### 3.3.1. Venturi Pump Interface—Mass Spectrometry

Atmospheric pressure chemical ionisation–mass spectrometry experiments were performed on an Advion Expression L compact mass spectrometer equipped with a commercial volatile APCI (vAPCI) source. The DOS probe arrangement, described in [27], was coupled directly to the vAPCI source heated line, enabling the direct desorption and extraction of volatilised species using a Venturi pump. The mass spectrometer conditions were as follows: capillary temperature 300 °C, capillary voltage 150 V, source voltage offset 25 V, source voltage span 30 V, source gas temperature 300 °C, transfer line temperature 100 °C, APCI corona discharge 4 µA. All experiments were performed in positive mode vAPCI. Thermal desorption was conducted at a temperature of ~60 °C using a heated gas flow rate of 0.5 L/min; the APCI gas flow was set to 4 L/min, which generates a suction flow of ~0.6 L/min. An acetone-d6 permeation source was present in line with the heated gas flow to act as an internal standard if required.

#### 3.3.2. IMS Conditions

The IMS system used in all IMS experiments in this study was a configurable Advanced Ion Mobility Spectrometer (AIMS) (MasaTech, Bratislava, Slovakia). The instrument parameters were set as follows: drift gas flow 700 mL/min, AIMS drift tube temperature 100 °C, sample inlet temperature ~90 °C, AIMS drift tube pressure 600 mbar. The IMS was operated in positive mode, with the corona discharge set at 11.5 kV and the target electrode set at 8.5 kV. The measurement period was set at 150 ms and the acquisition time was set at 300 s. The IMS was calibrated using 2-6-Di-tert-butylpyridine (2-6-DtBP), adjusting the drift-tube length within the software to obtain the mobility reference value of 1.422 cm^2^/Vs.

#### 3.3.3. Venturi Pump Interface—Ion Mobility Spectrometry

Initially, IMS experiments were performed using a modified version of the prototype desorption off surface (DOS) probe, developed by Rankin-Turner and colleagues [27], which was interfaced directly to the AIMS instrument using an in-house-constructed Venturi jet pump via a hollow tube split inlet, which allowed the flow exiting the Venturi pump to pass over the inlet capillary before being exhausted (Figure 2a). A flow of 4 L/min passing through the jet pump enabled a suction flow of 0.6 L/min to be extracted. From this, 0.2 L/min was sampled by the AIMS. The remainder of the flow was exhausted through the split inlet. The probe tip is held over the target at approximately 1 cm distance to facilitate thermal desorption.

#### 3.3.4. Microchamber Thermal Extractor Configuration—Ion Mobility Spectrometry

A microchamber thermal extractor interface (Markes µ-CTE250 microchamber thermal extractor (Markes International, Llantrisant, UK)) was supplied with a regulated flow of nitrogen gas (Figure 2b). A ¼″ o.d. stainless steel transfer line was interfaced directly to the sample port on the top of the microchamber using a Markes International tube interface cap. This was connected to a stainless-steel toggle valve to allow the flow to be switched on and off and to allow volatiles to build up in the microchamber headspace. The flow control of the microchamber was set to allow a flow of 600 mL/min of nitrogen when the valve was in the open position. Only a single microchamber was used in the analysis; the other 3 microchambers on the µCTE250 were blanked off to prevent the loss of gas flow. The interface was connected to the Masatech AIMS system using the same hollow tube split inlet described above.

#### 3.3.5. Dual-Disc Pump–Probe Interface—Ion Mobility Spectrometry

Two miniaturised ttpventus BL-P2-031 disc pumps (Lee Products Ltd., Chalfont St Peter, UK) were used to provide a pulsation-free gas flow. One pump was set up to draw filtered ambient air from the environment, which was heated to 60 °C by the cartridge heater in the body of the probe. Both pumps ran at a peak flow of 1.65 L/min. The heated air flow was then directed towards the sample from the tip of the probe at a distance of approximately 1 cm, initiating the desorption of volatile organic compounds (VOCs) from the sample’s surface. An adjacent transfer line captured the VOCs generated and directed them to the IMS inlet, powered by a second disc pump (Figure 2c). For the replicate analysis of biofluids and interferences, the acquisition time was 20 s. A blank sample of ambient air was acquired in-between each sample to ensure no sample carryover between analyses.

### 3.4. Data Processing

Classification was carried out within the Chemometrics data processing package built into the MasaTech software. A training subset of 80% of the data was used to train the model on each of the biofluids analysed, using the ‘averaged spectrum’ function. The test subset was composed of the remaining 20%. Principal component analysis (PCA) was then performed on the test set, again using the ‘averaged spectrum’ function, with the number of components set to 500 and by using a ‘Quic’ decomposition method. The data were then classified using Random Forest as the classification method. PCA plots could then be generated within the Chemometics software. The PCA .csv datafiles were interrogated in the in-built Chemometrics package, and data were then exported into Microsoft Excel to enable detailed visualisation. PCA plots were then generated by plotting individual components against each other using the ‘scatterplot’ function, which enabled increased functionality. The final plots were generated using a Python script and MatPlotLib [28]. The confidence ellipses represent 3 standard deviations from the mean. The Python script for this is available online [29].

## 4. Conclusions

Existing presumptive biofluid testing has many disadvantages, as outlined above. A reliable portable analytical device would eliminate many of these drawbacks. This initial proof-of-concept study has, for the first time, demonstrated the feasibility of using a thermal desorption probe combined with IMS to enable the reliable and potentially portable analysis of biofluids and common interferences in a controlled laboratory environment. The probe interface has several advantages for in situ analysis. It can analyse very small traces (single droplets of biofluid). Additionally, the gentle heating is non-destructive to the sample, and will allow subsequent analysis such as DNA profiling to be performed. The device also allows rapid analysis with minimal memory effects or carryover. In contrast to the microchamber configuration, it does have the drawback of lower sensitivity as fewer peaks are detected. However, further modification of the probe tip to allow it to seal over the target surface and reduce potential losses to the environment could mitigate this effect. The probe interface itself could also be expanded, if combined with an alternative light source (ALS) and camera; a single device could streamline the ‘visualisation → image capture → presumptive testing’ workflow that has previously been three separate and lengthy processes. It is envisaged that this could speed up the triaging of biofluid evidence encountered at a crime scene, accelerating the submission for further, confirmatory testing such as tandem mass spectrometry (MS/MS) or DNA profiling, depending on the intelligence required.

### Future Work

Future work will comprise expanding the number of biofluids investigated to include semen, saliva, sweat, vaginal fluid and menstrual blood. In addition, additional substances commonly recognised to provide erroneous false-positives to conventional presumptive testing kits will be examined. Finally, it is acknowledged that this proof-of-concept experiment has been performed in a laboratory environment, without many of the contaminants and environmental conditions that may be experienced in situ in the field. A thorough investigation into the robustness of the technique will be appraised in various environments, surfaces and with samples of various ages. The surface on which desorption is conducted from, in particular, will influence the data obtained; surfaces may emit volatiles which influence the profile obtained, or an absorbent surface may retain VOCs, so a systematic investigation of different surface types is required. While this is a significant issue, strategies such as conducting an analysis of the surface background and then performing a background subtraction can be used to compensate for differences between samples using DOS probe analysis and will be explored as potential mitigation strategies. In addition, this technique will also be evaluated using mixed samples of more than one substance type (multiple mixed biofluids, biofluids mixed with interferents) to better replicate those found in situ at crime scenes.

## Figures and Tables

**Table 1:** Prominent mobility value signals present in each of the samples analysed.

Substance	Prominent Mobility Value Signals (cm^2^/Vs)
Blank	1.82, 1.92, 1.96, 2.19, 2.44
Blood	1.80, 2.42
Urine	1.60, 1.82, 2.42
Ketchup	1.60, 1.82, 1.90, 2.06, 2.18, 2.42
Food colouring	1.64, 1.82, 1.90, 2.08, 2.44
Shoe polish	1.56, 1.62, 1.82, 1.90, 2.42

## Data Availability

Data are available in a publicly accessible repository that does not issue DOIs; publicly available datasets were analysed in this study. These data can be found here: [https://www.lboro.ac.uk/research/support/publishing/institutional-repository] (accessed on 5 September 2023).

## Supplementary Materials

The following supporting information can be downloaded at https://www.mdpi.com/article/10.3390/molecules28186533/s1, (https://clipchamp.com/watch/cFFU5RUVBE6 accessed on 15 June 2023), Figure S1: Ion mobility heat map obtained from a 10 μL dried human saliva spot obtained using the microchamber thermal extractor configuration showing mobility versus time, indicating switching between background and dried blood spot profiles. Figure S2: Ion mobility data from a 10 μL dried urine spot obtained using the microchamber thermal extractor configuration showing mobility versus time, indicating switching between background and dried blood spot profiles. Video S1: Real-time acquisition of ion mobility data from the IMS analysis of samples using the DOS probe.
