# Supplementary material for: Differentiation of Body Fluid Stains Using a Portable, Low-Cost Ion Mobility Spectrometry Device—A Pilot Study
Source: Molecules. 2023 Sep 9;28(18):6533. doi: 10.3390/molecules28186533 (PMC10534372; doi:10.3390/molecules28186533)
Supplement: Supplementary file 1 [file molecules-28-06533-s001.zip › molecules-2535282-supplementary.pdf]

Differentiation of body fluid stains using a portable, low-cost Ion Mobility Spectrometry device – A pilot study

Electronic Supplementary material

Cameron Heaton [1,2*], Simon Clement[1], Paul F. Kelly[2], Roberto S. P. King[1] and James C. Reynolds[2]

[1] Foster + Freeman; cameron.heaton@fosterfreeman.com
[2] Department of Chemistry, Loughborough University, Loughborough, LE11 3TU; c.heaton@lboro.ac.uk
* Correspondence: roberto.king@fosterfreeman.com / j.c.reynolds@lboro.ac.uk

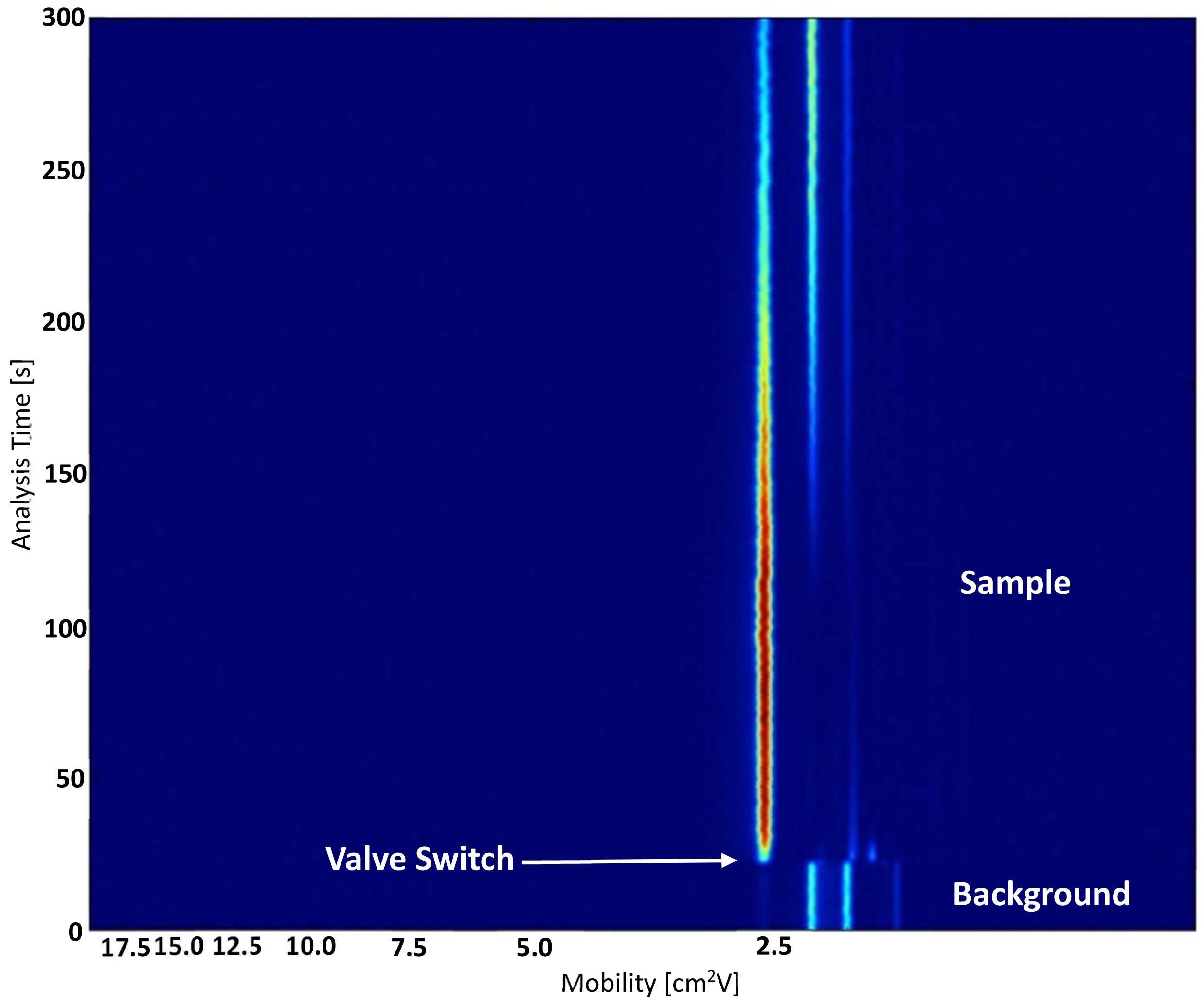

Figure S1 – Ion mobility heat map obtained from a 10 μl dried human saliva spot obtained using the microchamber thermal extractor configuration showing mobility versus time indicating switching between background and dried blood spot profiles.

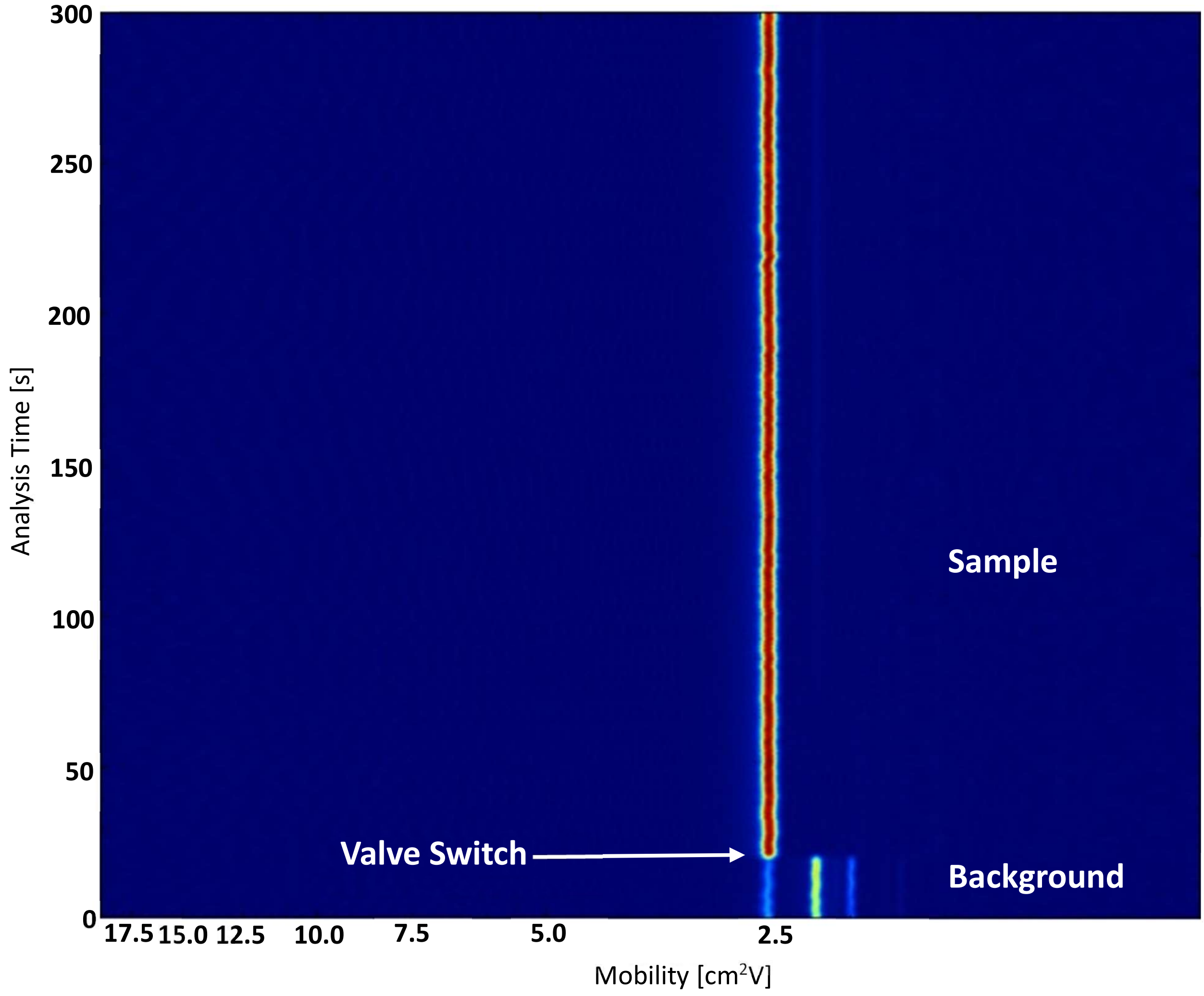

Figure S2 – Ion mobility data from a 10 µl dried urine spot obtained using the microchamber thermal extractor configuration showing mobility versus time indicating switching between background and dried blood spot profiles.
